# Distribution and Feeding Behavior of *Omorgus suberosus* (Coleoptera: Trogidae) in *Lepidochelys olivacea* Turtle Nests

**DOI:** 10.1371/journal.pone.0139538

**Published:** 2015-09-30

**Authors:** Martha L. Baena, Federico Escobar, Gonzalo Halffter, Juan H. García–Chávez

**Affiliations:** 1 Instituto de Investigaciones Biológicas, Universidad Veracruzana (IIB–UV), Xalapa, Veracruz, México; 2 Instituto de Ecología, A. C., Red de Ecoetología, Xalapa, Veracruz, México; 3 Laboratorio de Ecología de Poblaciones, Escuela de Biología, Benemérita Universidad Autónoma de Puebla, Puebla, México; James Cook University, AUSTRALIA

## Abstract

*Omorgus suberosus* (Fabricius, 1775) has been identified as a potential predator of the eggs of the turtle *Lepidochelys olivacea* (Eschscholtz, 1829) on one of the main turtle nesting beaches in the world, La Escobilla in Oaxaca, Mexico. This study presents an analysis of the spatio–temporal distribution of the beetle on this beach (in areas of high and low density of *L*. *olivacea* nests over two arrival seasons) and an evaluation, under laboratory conditions, of the probability of damage to the turtle eggs by this beetle. *O*. *suberosus* adults and larvae exhibited an aggregated pattern at both turtle nest densities; however, aggregation was greater in areas of low nest density, where we found the highest proportion of damaged eggs. Also, there were fluctuations in the temporal distribution of the adult beetles following the arrival of the turtles on the beach. Under laboratory conditions, the beetles quickly damaged both dead eggs and a mixture of live and dead eggs, but were found to consume live eggs more slowly. This suggests that *O*. *suberosus* may be recycling organic material; however, its consumption of live eggs may be sufficient in some cases to interrupt the incubation period of the turtle. We intend to apply these results when making decisions regarding the *L*. *olivacea* nests on La Escobilla Beach, one of the most important sites for the conservation of this species.

## Introduction

The olive ridley turtle (*Lepidochelys olivacea*) is a pantropical species common in the Pacific, Indian and southern Atlantic oceans, where its major nesting beaches and foraging areas are found [1]. One of the most important nesting areas of this turtle in the eastern Pacific is La Escobilla Beach in Oaxaca, Mexico [2]. The high abundance of the beetle *Omorgus suberosus* recorded on this beach over the last fifteen years is of great concern, since this beetle appears to be capable of consuming viable turtle eggs. It is therefore considered another risk factor for the survival of this species of turtle [3–4].

**Fig 1:**
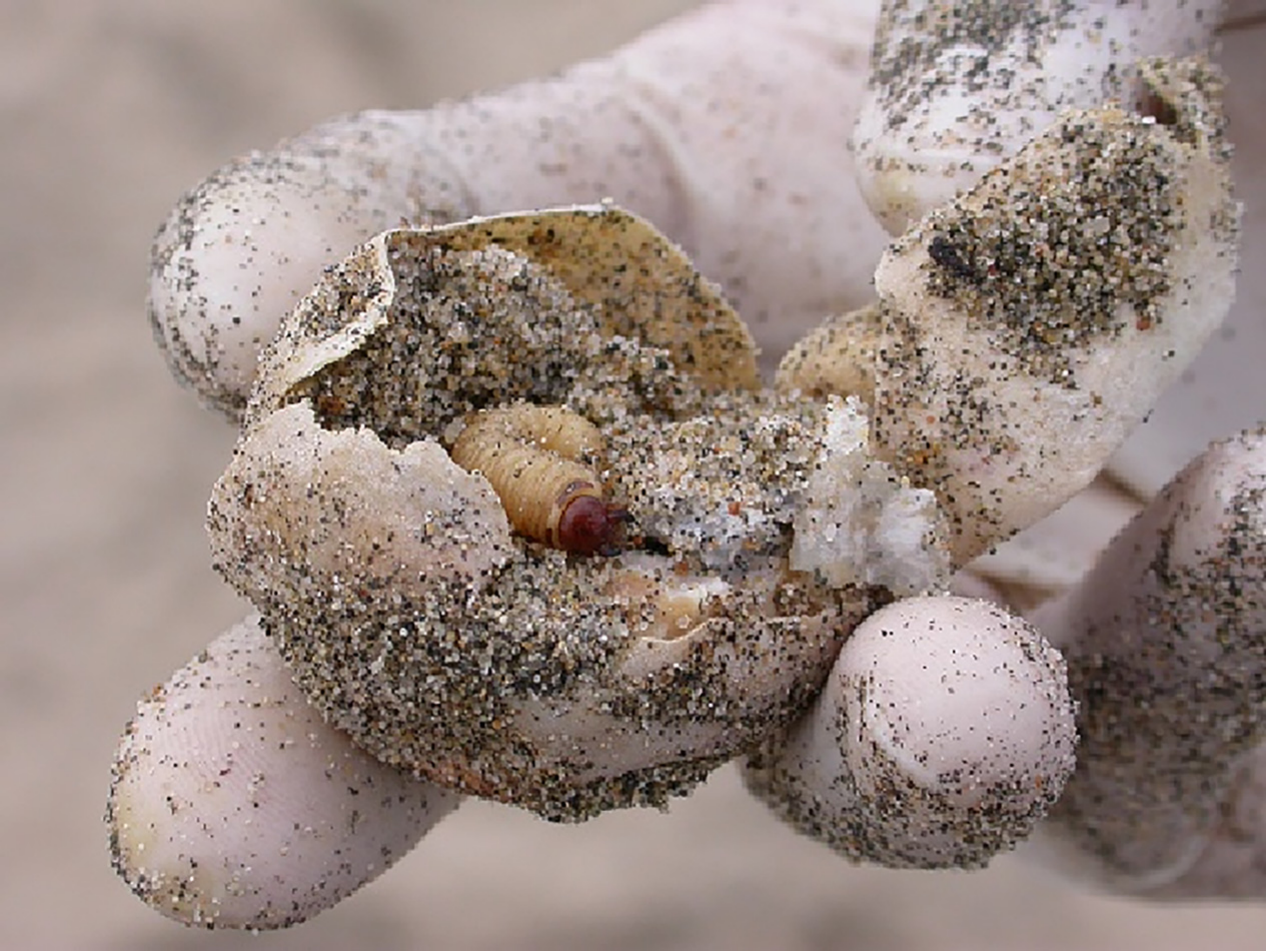
Larva of *Omorgus suberosus* feeding on the eggs of *Lepidochelys olivacea* on La Escobilla Beach, Oaxaca, Mexico.

This beetle has been reported to enter *L*. *olivacea* nests on other beaches (e.g., Ostional Beach, Costa Rica) as well as the nests of other sea turtle species [4], such as *Chelonia mydas agassizi* Linnaeus in the Galapagos Islands, Ecuador. At the latter, iguana eggs (*Conolophus subcristatus* Gray) buried in the sand have also been depredated by *O*. *suberosus* [4–5]. Furthermore, it has been reported to be a predator of the eggs of the migratory locust *Schistocerca paranensis* Burmeister in Argentina [6]. Recently, and confirmed by this study, the predation of *L*. *olivacea* eggs was observed when larvae and adult of the beetle consumed their shells and yolk on La Escobilla Beach (Fig 1). While it is thought that *O*. *suberosus* on La Escobilla Beach could increase the mortality of *L*. *olivacea* embryos and hatchlings [4, 7], there is no evidence to date of this beetle affecting the survival of this turtle’s young.

The presence of *O*. *suberosus* in olive ridley turtle nests on La Escobilla Beach is related to the massive and synchronized arrival of females of the turtle (called arribadas). Female turtles emerge from the sea to lay their eggs in the sand for a few consecutive nights over a period of up to several weeks [8]. Using the Gates–Valverde transect count method, a government research program estimated there were *ca*.1.2 million nests in 2009–2010 at La Escobilla [9], making it one of the largest nesting populations in the world [7]. Even so, *L*. *olivacea* is listed on the International Union for the Conservation of Nature (IUCN) Red List as vulnerable [10]. Turtle survival is thought to be low on high density nesting beaches because of density–dependent mortality [11] which leads to hatchling rates as low as 1 to 8% [12]. Other factors that affect the survival of the turtle include intra–specific nest destruction, i.e. damage to previously laid eggs by other females that arrive later during an arribada [8], removal by humans, microclimatic variation in the nests, and the predation of eggs and young by vultures, dogs and crabs [1, 13–14]. To date, however, the impact of these mortality factors on *L*. *olivacea* is unknown [7].

It has been recently proposed that the high density of nests of the turtle have favored an increase in the population of *O*. *suberosus*. On the other hand, in the presence of a plentiful food supply of any type of food, this beetle appears to behave as a predator, which could affect the survival of this turtle [3, 15]. This suggests a change in feeding behavior, since the adults and larvae of this beetle are normally necro–saprophagous in habit, consuming feathers, hair, skin and bone in the final stages of decomposition [16–21]. On finding food, female beetles bury themselves in the soil and oviposit close to the food source. On emergence, the larvae feed on the animal remains and stay buried until the adult stage when they move to the soil’s surface to mate [20–22].

Food availability is one of the ecological factors that regulate the spatial and temporal distribution of organisms [23–24]. It is an essential part of population dynamics because it can affect the behavior of individuals within the population as well as the demographics and relationship of organisms with the habitat [23, 25–28]. The description and explanation of the spatial and temporal distribution patterns of individuals are therefore important in the development of monitoring programs for estimating population size and managing pests [27, 29–30]. Our objective was to examine the spatial distribution of the beetle in the field relative to olive ridley sea turtle nest density and the temporal distribution of this beetle relative to the arrival of female turtles on La Escobilla Beach.

Based on our field observations regarding the presence of *O*. *suberosus* in turtle nests that contain both viable live and dead eggs, we also assessed whether consumption is a function of the condition and abundance of turtle eggs, factors that may influence the total time invested by the beetle in searching for and consuming food. We also documented the life cycle of *O*. *suberosus* in order to analyze the relationship between the stages of beetle development and the incubation of turtle eggs.

To our knowledge, no study has yet analyzed the spatial and temporal dynamics of *O*. *suberosus*, nor has the relationship between the frequency of this beetle and turtle egg damage been documented under field conditions. Furthermore, the feeding behavior of the beetle on eggs of *L*. *olivacea* has not been evaluated in the laboratory. Based on the hypothesis that food quantity influences the feeding behavior and distribution patterns of *O*. *suberosus* on La Escobilla Beach, our predictions were: 1) a high frequency of beetles within the turtle nest would influence the proportion of damaged eggs, especially in sites with high nest density, and 2) the beetles would be distributed in an aggregated manner on sites with low turtle nest density where food availability is lower. Considering the necro–saprophagous habit of *O*. *suberosus*, under laboratory conditions we tested the hypothesis that the abundance and condition of the eggs would affect the probability of their being consumed within a given period of time and made two predictions: 1) the probability of all the eggs in a single clutch being consumed over a shorter period of time will be greater for dead than for live eggs, and 2) when eggs are less abundant they will be consumed more quickly.

With the results of this study, we intend to contribute to informed decision–making regarding turtle nest management, a very important aspect of the conservation of *L*. *olivacea* on La Escobilla Beach.

## Materials and Methods

### Study area

The turtle sanctuary of La Escobilla Beach (hereafter La Escobilla) is located in the municipality of Santa María Tonameca, in the state of Oaxaca, Mexico (15° 47' N; 96° 44' W). The sanctuary was created in 1986 to protect the olive ridley sea turtle and is managed by the National Commission of Protected Natural Areas (CONANP, is its acronym in Spanish). The weather is warm–subhumid (Aw0) with a mean annual temperature of 28°C and mean annual precipitation of 1,000 mm, most of which falls from May to October [31]. The turtles nest along an eight-kilometer-long stretch at the western end of the beach, which is approximately 22 kilometers long. According to Sarti et al. [32], clutches are deposited across the width of the beach in three ecological zones: 10% are deposited in the intertidal zone (zone A), 75% in the middle of the beach (berm and platform) (zone B) and 15% in the littoral vegetation (zone C). To facilitate monitoring both the turtle arrivals and nests, the Mexican Turtle Center–CONANP has divided the most frequent nesting areas on the beach into 160 stations, marked every 50 m by a post (8 km in total). This system made it possible to identify the location of the sites of high and low density of turtle nests and to place the beetle sampling traps along this part of the beach.

### Ethics Statements

This research was fully funded by the National Commission for Protected Natural Areas (CONANP–Mexico). Both the field and the laboratory experiments of this study were planned in conjunction with the respective authorities. The Direction of Species of Conservation Priority of CONANP authorized the collection of nests from the beach, as well as the transportation and manipulation of turtle eggs and freezing the eggs in order to conduct the laboratory experiments (OFICIO NÚM. SGPA/DGVS/03017/08, 21 MAY 2008).

### Abundance and spatial distribution of *O*. *suberosus* at low and high *L*. *olivacea* nest densities

*O*. *suberosus* is more abundant on sites of the beach between the foreshore and the vegetation (zone B), where the highest turtle nesting activity is concentrated [33]. With this in mind and based on the recommendations of staff of the Mexican Turtle Center–CONANP in charge of the turtle monitoring program, we selected sites of high and low turtle nest density in order to evaluate the abundance and distribution pattern of *O*. *suberosus*. For each density, we counted all of the nests buried in the sand at each sampling unit (details below). This method has also been used to determine sites of low and high nest density on this beach [7].

For each nest density, we randomly selected 20 sampling points separated by at least 20 m. At each sampling point, an area of 1 m^2^ (sampling unit) was delimited using a quadrat made from plastic PVC tubing, for a total sampling area of 20 m^2^ per density (40 m^2^ in total). The quadrat was used because a) turtle nests can be contiguous with those of other turtles, and even on top of other nests, so it is possible to find more than one nest per quadrat, and b) it is a useful method to evaluate the distribution of organisms that live in relatively continuous habitats [26, 28], as is the case with this beetle species on La Escobilla.

Quadrats were sampled between 07:00 h and 14:00 h, since the turtles lay their eggs during the night (regularly between 22:00 h and 06:00 h) [34]. In order to find the nests within each quadrat, a 1 m wooden pole was pushed down into the sand every 10 cm over the entire 1 m^2^ area. When the pole went through the sand with little resistance, this indicated the presence of a nest below (at 30 cm depth on average, Harfush pers. comm.). Once a nest had been located, a shovel was used to remove the sand from above it and the eggs were manually removed. For overlapping nests, we used a count of 100 eggs (the average value of each clutch size) as the criterion to determine the number of nests in each sampling unit [35]. To quantify the number of larvae and adult beetles above, in and around the nest, the sand was sieved through a 0.5 cm^2^ mesh and the insects were counted. For each quadrat, the following variables were quantified: 1) the number of nests / m^2^, 2) the number of eggs / nest / m^2^, 3) the number of *O*. *suberosus* (adults and larvae) / nest / m^2^, and 4) the number of eggs perforated by beetles / nest (Fig 1).

Sampling was conducted over two arrival seasons: during the 6^th^ arribada of the 2008–2009 season (17–23 September 2008), which was one of the biggest of the season, and at the beginning of the 2011–2012 season (24–27 May 2011) after the first arrival had finished, which was one of the smallest of the season. It is noteworthy that an arribada lasts 7–10 months. Every arrival season has several (between 6 and 9) turtle arrival events, each of which lasts 4–17 days. During the 2008–2009 season, there were eight arrival events (start: June 29, 2008; end: January 21, 2009), while in the 2011–2012 season, there were six (start: May 2, 2011; end: February 4, 2012).

### Temporal distribution of *O*. *suberosus* on La Escobilla

Since the adult beetles were active on the surface of the beach, especially at night, and with the aim of evaluating the temporal distribution patterns of *O*. *suberosus* on the beach, we sampled over a period of 35 weeks (September 2010 to June 2011) along a stretch of approximately 2 km of beach where *L*. *olivacea* builds nests. Forty-one stations were selected, separated by 50 m, and three beetle traps were set on the beach at each station, for a total of 123 traps. Sampling spanned turtle arrival events 3 to 6 of the 2010–2011 season, the first two of season 2011–2012 and the period between seasons.

Each trap consisted of an 80 L plastic container (40 x 40 x 50 cm) buried in the sand flush with the ground, half–filled with water and with 15 g of an odorless detergent to prevent the trapped beetles from escaping. Each container was covered with a 2.5 cm^2^ mesh that allowed the beetles to enter the trap. As the attractant, 300 g of chicken feathers were placed on the mesh and tied down with rope to prevent removal by the wind. The feathers were dampened with water every two or three days to maintain their humidity and olfactory attractiveness to the beetles and replaced with new feathers every six days. Trapped beetles were removed daily in the morning and the liquid in the traps was changed every 20 days.

### The effect of the condition and abundance of *L*. *olivacea* eggs on the feeding behavior of *O*. *suberosus*

A laboratory experiment was conducted (environmental conditions: temperature: 28°C, relative humidity: 75%, illumination: 12 h light/day) to estimate the probability of turtle eggs being consumed by the beetles, taking two factors into account: 1) egg condition, with three levels: 100% live eggs (with apparent development, hereafter referred to as live), 50% dead and 50% live eggs (hereafter referred to as combined) and 100% dead eggs (hereafter referred to as dead) and 2), the abundance of the eggs (low and high). For the experiment, six treatments were used, maintaining a fixed ratio of beetles to eggs in each treatment (2.5 beetles / egg), in order to control relative beetle density. Each treatment had five replicates, each of which consisted of plastic boxes (43 x 31 x 17 cm), which were considered the experimental unit (EU). The treatments were: T1: low number of live eggs (8 x 5 = 40 eggs with 100 beetles); T2: high number of live eggs (16 x 5 = 80 eggs with 200 beetles); T3: low number of dead eggs (8 x 5 = 40 eggs with 100 beetles); T4: high number of dead eggs (16 x 5 = 80 eggs with 200 beetles); T5: low number of combined eggs (8 x 5 = 40 eggs with 100 beetles); T6: high number of combined eggs (16 x 5 = 80 eggs with 200 beetles). A total of 360 eggs and 900 adult beetles were used in this experiment.

Each box (EU) was filled to three–quarters with sand from La Escobilla from which all organic matter had been removed. The eggs were placed in a hollow in the sand, simulating the manner in which the turtles oviposit under natural conditions, without fully covering them. The contents of the box were sprayed with potable water every third day to keep the sand damp. The live turtle eggs (after approximately one week of incubation) were recognized as such by their white color and turgidity, and the beetles used in the experiment were transported carefully from La Escobilla to the Experimental Ecology Laboratory at the Instituto de Ecología, A. C., in Xalapa, Veracruz. The eggs were transported in a plastic box (80 x 30 x 50 cm), covered with vermiculite to keep them moist and prevent damage. Beetles were transported in a plastic box (25 x 15 x 10 cm) with wet sand. To obtain dead eggs, live eggs were frozen –20°C for 24 h, removed from the freezer and left at room temperature for 12 hours to bring them to the same temperature as the live eggs. In the treatment with a combination of live and dead eggs, the latter were marked with indelible ink for identification. To determine the extent of the damage caused by the beetles over time, all eggs were checked daily.

An egg was considered damaged if its shell was perforated. Prior to the experiment, we determined that egg damage is initiated by the beetles rasping the surface with their mandibles until they break through, resulting in small perforations (0.03–4.8 cm^2^). Two or three days later, the contents of the egg begin to solidify. At this stage, the beetles eat their way into the egg until they have consumed all of it, leaving only fragments of the shell.

### Life cycle

In plastic boxes (25 x 15 x 10 cm) filled to three–quarters of their capacity with beach sand, we placed ten adult beetles with one turtle egg and waited 10 to 15 days to obtain immature stage beetles, which were individually placed in new plastic boxes with a turtle egg. Every third day, the boxes were checked to record changes from one stage to another until adulthood was reached. The environmental conditions in the laboratory for this experiment were the same as those described above.

### Data analysis

#### Abundance and spatial distribution of *O*. *suberosus* at low and high *L*. *olivacea* nest densities

To determine whether the proportion of *L*. *olivacea* eggs damaged is a function of the frequency of *O*. *suberosus* in both turtle nest densities, we applied an analysis of covariance using the proportion of damaged eggs as the response variable and density (two levels: high and low) as the explanatory variable. The frequency of adult beetles was included as a covariable. We applied a generalized linear model with a binomial distribution and the logit link function [ln (*p*/*q*, where *p* and *q* are the proportions of damaged and undamaged eggs, respectively)]. Where the fit of the data to the statistical model indicated overdispersion, the model was adjusted with the quasi–likelihood method [36].

In order to determine whether the frequency of larvae is a function of the frequency of *O*. *suberosus* adults, we used an analysis of covariance run as a generalized linear model, with a Poisson distribution and using the log link function, where the response variable was the number of larvae. Nest densities in 2008–2009, with two levels (high and low density), were included in the model as the explanatory variable and the frequency of adult beetles as a covariable. The quasi–likelihood method was fitted when there was overdispersion [36]. Data for these variables from 2011–2012 were not used, because no larvae were found during that season (see S1 Table).

*O*. *suberosus* distribution patterns at sites of high and low turtle nest densities from the samples taken in 2008–2009 and 2011–2012 were estimated using the Mean Crowding index (m*), which relates spatial distribution to density–dependent behavior. In biological terms, this index is useful for analyzing the spatial distribution of organisms in natural populations in relation to the ecological effects of crowding and competition, because it is based on the principle that individuals in wild populations are aggregated, since a random distribution would imply the absence of any biological behavior. Additionally, it is theoretically independent of the scale of sampling and produces reliable results for quadrat samples that fit a negative binomial distribution [26].

Mean Crowding (m*) measures the mean distance to the nearest neighbor. Therefore, it is the degree to which individuals tend to be clumped on resource patches (the larger the value of m*, the greater the degree of aggregation of individuals on resource patches) and it is calculated as: m* = *m* + [(*V*_*m*_/ *m*) − 1]. This is the sum of the mean density, representing the population, and the variance ratio, representing the crowding effect, less one, which is the individual in the quadrat [30]. In this study, the mean density of beetles is: *m* = *Σ m*_*i*_/*M*, where *m* was calculated in two ways: as the average density of beetles / m^2^ and as the average density of beetles / nest, where *m*_*i*_ = the number of beetles and M = total number per m^2^ and per nest. *Vm* is the variance in the number of beetles and in each case is calculated as: *Vm* = [(Σ *m*_i_)^2^/ *M*] − (*m*)^2^. When the *Vm* > *m*, then individuals are clumped in space and m* is greater than the arithmetic mean density (*m*). If *Vm* < *m*, then individuals are overdispersed and m* is relatively smaller than *m*. When *Vm = m* then individuals are randomly dispersed and m* is equal to *m*.

To express the degree of nonrandom aggregation on resources, patchiness (***P***) is an index of the relative concentration of individuals on resources [26]. The value of ***P*** is given as: ***P*** = m* / *m*. Hence, this index describes this relationship as “how many times as ‘crowded’ an individual is, on the average, as it would have to be if the same population had a random distribution” [26]. When patchiness ***P*** > 1 individuals are aggregated around certain clusters of resources. When ***P*** < 1, individuals are distributed more evenly over resources than would be expected by chance [26]. Lloyd [26] mentioned that mean crowding and patchiness (m* / *m*) only have meaning when the population is loosely aggregated and the quadrat size is not too large as compared with the ambit of an individual.

#### Temporal distribution of *O*. *suberosus* on La Escobilla Beach

We used Shuster & Wade’s [37] adaptation of the index of Mean Crowding (described above) to analyze the temporal distribution (of beetle captures in traps) over 35 weeks during season 2010–2011. Mean temporal crowding is: t* = *t* + [(*V*_*t*_/*t*) − 1] and the average number of beetles per interval of time is calculated as: *t* = *Σt*_*i*_/*T*, where *t*_*i*_ = the number of beetles and *T* = the total number of time intervals (in this case, 7 time intervals each of 5 weeks, n = 35 weeks). The variance in the number of beetles is calculated as: *V*_t_ = [(Σ *t*_i_)^2^ / *T*] − (t)^2^. Temporal patchiness is: ***S*** = t* / *t*. The assumptions of mean temporal crowding are similar to those of mean spatial crowding. A bootstrap resampling procedure (10,000 resamples) was used to estimate the 95% Confidence Intervals of the parameters described above. The CI value of 95% was used to compare ***P*** values found in high and low densities in each arrival season (2008–2009 and 2011–2011) and ***S*** for each five week period during season 2011–2012. The analysis was run in R.12.2 [38] in combination with the ‘boot’ library [39].

#### The effect of condition and abundance of *L*. *olivacea* turtle eggs on damage by *O*. *suberosus* beetles

We evaluated two biological aspects. First, we documented the time that elapsed from the beginning of the experiment until the first egg of each experimental unit (EU) was damaged by an *O*. *suberosus* adult (time 1). This is important because if damage begins when the eggs have been recently laid, the entire EU will be at greater risk of being completely consumed than in those nests where damage is initiated at a later time. Second, the time it takes for the EU to be completely consumed (time 2). This indicates the probability that the EU ends up with at least one egg intact. To this end, we recorded two events (the damage to the first egg and the consumption of the entire nest) as a function of time in days. It should be noted that we do not record percent damage, or the final destiny of individual eggs because this does not provide accurate information about these two events.

In order to determine the probability of an egg being consumed as a function of time and its association with the two factors of interest (the condition of the egg and egg abundance), a survival analysis was run with a time regression model [36, 40–41]. We use an accelerated failure time model because these are more robust than the proportional hazards models traditionally used in studies of human medicine [41]. This analysis allows us to compare the probability curves for the occurrence of the event of interest (i.e., damage to the first egg or damage to all of the eggs in each experimental unit) over time, as a function of the independent variables. The complete statistical model included the effect of the abundance of eggs (with two levels: high and low) and egg condition (with three levels: live, dead and combined) and their interaction. The complete statistical model was simplified following the criteria of [36]. Cases in which the experimental units did not contain any damaged eggs (time 1) or in those in which some eggs remained unconsumed (time 2), i.e., in which the event of interest was not recorded, were considered censored data. We assumed a Weibull error distribution because it does not require damage to the eggs to be constant over time [36]. The analysis was run in R freeware v.2.12.2 [38] and in the Survival package [42].

## Results

### Abundance and distribution of *O*. *suberosus* at low and high *L*. *olivacea* nest densities

Turtle eggs were found buried at 20 to 35 cm in depth, together with beetle larvae and adults. A total of 73 turtle nests were examined in 2008–2009 and 85 nests were examined in 2011–2012, with 7,217 and 8,865 eggs, respectively (Table 1).

**Table 1:** Variables for each *L*. *olivacea* nest density over two arrival seasons on La Escobilla Beach, Oaxaca, Mexico. Data provided by the Centro Mexicano de la Tortuga–CONANP, Mexico for the sixth arribada 2008–2009 with an estimated 116,000 nests (~11.5 million turtle eggs). For the first arribada (2011–2012) about 4,476 nests (~447,000 eggs) were estimated.

Arrival season	2008–2009 (6^th^ arribada)		2011–2012 (1^st^ arribada)	
Density	Low	High	Low	High
**Nests / m** ^**2**^	1.6 ± 1.4 (32)	2.0 ± 1.6 (41)	1.4 ± 2.2 (29)	2.8 ± 1.8 (56)
**Eggs / m** ^**2**^	134.7 ± 132.7 (2694)	226.1 ± 187.3 (4523)	204.8 ± 169.4 (4097)	238.4 ± 213.6 (4768)
**Eggs / nest (clutch size)**	84.2 ± 33.8 (2694)	110.3 ± 49.6 (4523)	141.3 ± 39.4 (4097)	85.1 ± 69.3 (4768)

Mean ± SD. Total is shown in parenthesis.

**Fig 2:**
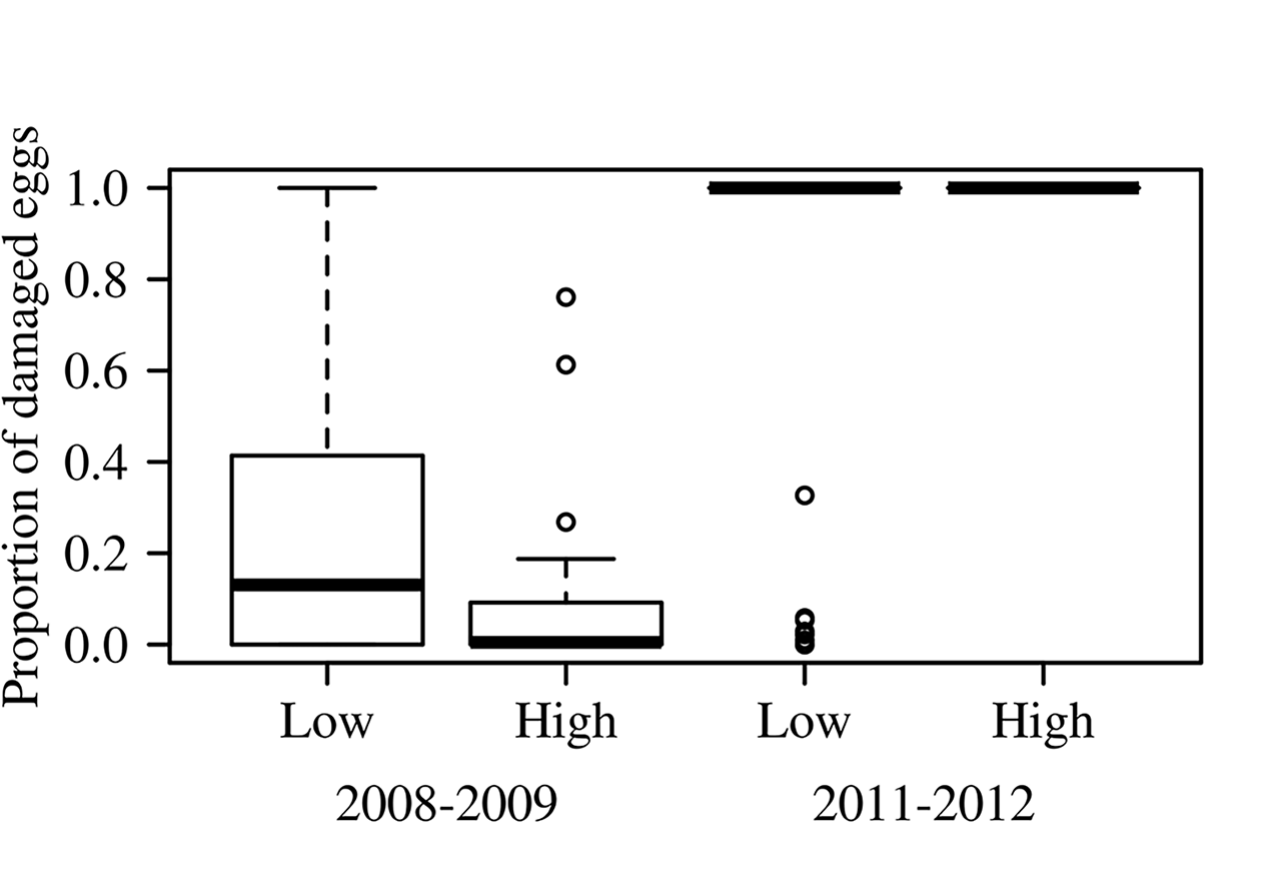
Proportion of *L*. *olivacea* eggs damaged at high and low nest densities on La Escobilla Beach, Oaxaca, Mexico, in the 2008–2009 and 2011–2012 arrival seasons. The line within each box represents the median, and the height of each box represents the first and third quartiles.

The proportion of damaged eggs differed between arrival seasons (*Chi* ^*2*^ = 9488.7, df = 1, p < 0.0001), being much higher in 2011–2012 (Fig 2). Contrary to our prediction, the analysis of covariance showed that the frequency of adult beetles as a covariable was not associated with the proportion of damaged eggs (*Chi* ^*2*^ = 149.3, df = 1, p = 0.083). With regard to the Y–intercept of each group, the contrast analysis showed that these differed from zero and also differed among themselves (*t* = 2.26, p = 0.027). Also contrary to expectation was the relatively lower proportion of damaged eggs that we recorded in the high nest density sites. The difference was marked by the proportion of damage found in the 2008–2009 season, while at the beginning of the 2011–2012 season in both densities, virtually all eggs had been damaged (*Chi* ^*2*^ = 956.8, df = 1, p < 0.001; Fig 2).

**Fig 3:**
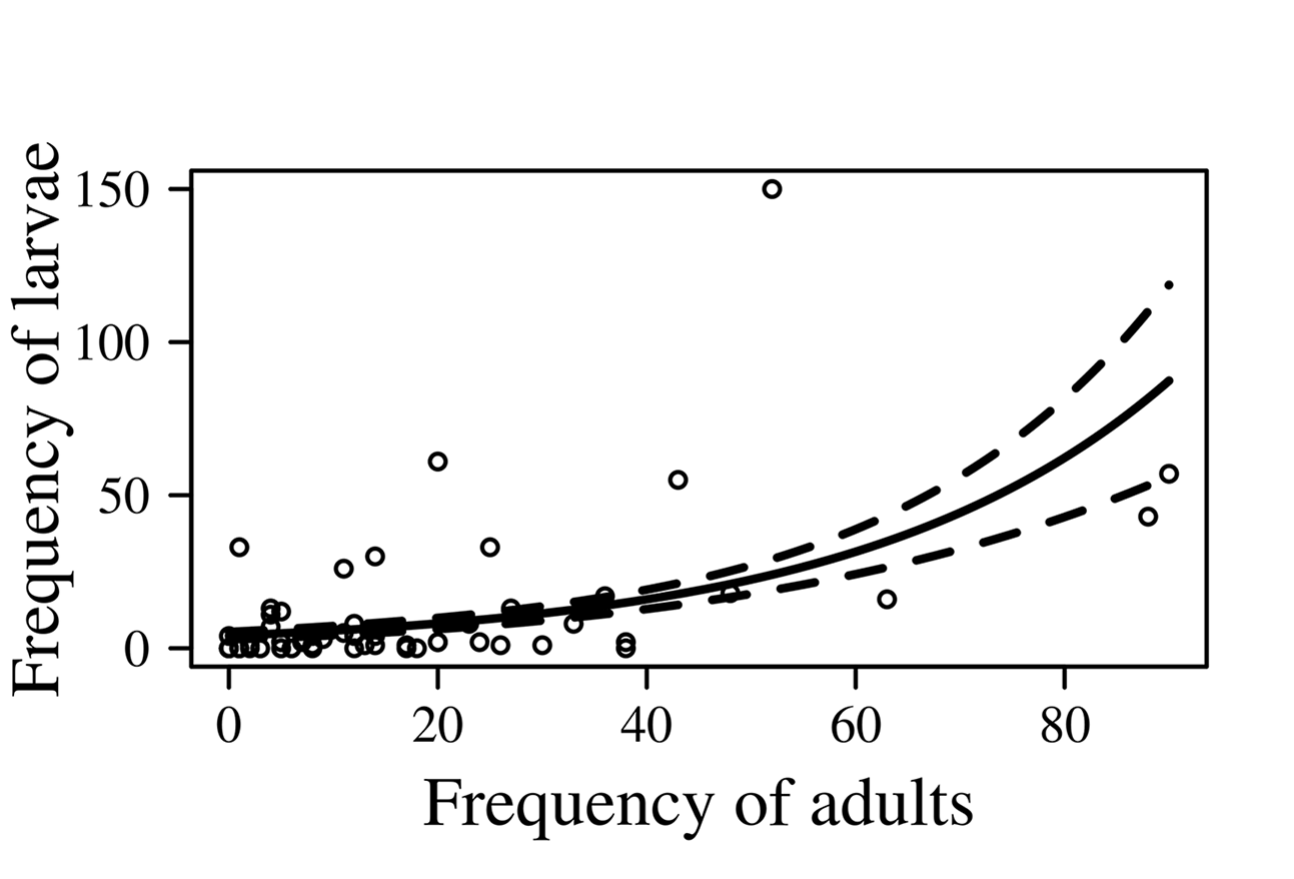
Relationship between the frequency of *O*. *suberosus* larvae and adults in *L*. *olivacea* nests on La Escobilla Beach, Oaxaca, Mexico. The solid line represents the values expected according to the ANCOVA. The dotted lines represent the standard error along the regression line.

We found that the frequency of adult *O*. *suberosus* in the turtle nests was related to the frequency of larvae (Fig 3); however, this was independent of nest density since, and according to the contrast analysis, neither the Y–intercept nor the slope of either density differed significantly (*t* = 0.629, *p* = 0.5 for the intercept; *t* = 1.08, p = 0.28 for the slope).

The distribution pattern of adult beetles in the quadrats and nests was aggregated during both arrival seasons and at both turtle nest densities (*Vm* > *m*, Table 2A and 2B). We found that the value of spatial patchiness (***P***) estimated for adult beetles / m^2^ at the low density of turtle nests in the season 2011–2012 was 2.6 times greater (ratio 5.8 / 2.2) and statistically different from that recorded at the high nest density, as indicated by the non–overlap of the 95% CI (Table 2A). For the same season, a similar pattern was found for adult beetles / nest: this was two times more aggregated at the low than it was at the high nest density, although no statistical differences were detected (Table 2B). Similarly, in the 2008–2009 season, we found that the spatial patchiness of adult beetles per m^2^ and per nest was greater in the low compared to high nest density but that these differences were not significant. However, larvae were 2.6 times (ratio 9.8 / 3.7) more aggregated in the low nest density in the 2008–2009 season, while in 2011–2012, the distribution of the larvae in the high density of nests had a distribution that fell between regular and random (***P*** = 0.98) (Table 2C).

**Table 2:** Parameters of the spatial distribution of *O*. *suberosus* adult beetles (2A and 2B) and larvae (2C over two arrival seasons on La Escobilla Beach, Oaxaca, Mexico.

**(2A) Adult beetles / m** ^**2**^					
**Arrival season**	**Density**	**No. ind.**	**Mean (*m*) (CI 95%)**	m* (CI 95%)	*P* (m*/ *m*) (CI 95%)
2008–2009	High	626	31.3 (20.1–47.7)	60.1 (41.9–90.9)	1.9 (1.5–3.1)
2008–2009	Low	467	23.3 (11.6–40.5)	66.2 (46.3–87.6)	2.8 (1.9–5.4)
2011–2012	High	702	35.1 (23.7–60.1)	76.6 (39.9–129.5)	2.2 (1.5–3.3)
2011–2012	Low	286	14.3 (5.5–40.7)	82.6 (23.4–132.9)	5.8 (3.4–15.7)
**(2B) Adult beetles / nest**					
2008–2009	High	626	13.0 (9.7–19.1)	30.7 (18.8–55.9)	2.3 (1.7–3.7)
2008–2009	Low	467	15.0 (8.8–24.5)	44.5 (29.7–66.8)	2.9 (2.1–4.7)
2011–2012	High	702	12.5 (9.4–17.2)	28.1 (20.2–38.1)	2.2 (1.8–2.9)
2011–2012	Low	286	9.9 (5.7–23.6)	43.3 (8.6–82.1)	4.4 (1.8–7.7)
**(2C) Larvae / nest**					
2008–2009	High	363	8.0 (4.5–13.3)	29.8 (16.2–44.3)	3.7 (2.6–5.6)
2008–2009	Low	287	9.6 (3.0–29.3)	93.6 (21.4–145.3)	9.8 (5.6–23.6)
2011–2012	High	7	1.7 (1.0–2.5)	1.7 (0.0–2.4)	0.98 (0.0–1.0)
2011–2012	Low	0	0.0 (0.0)	0.0 (0.0)	0.0 (0.0)

95% CI Bootstrap is given in parenthesis. Value of spatial patchiness (***P***) depends on m* and *m*. When m* = 1, then *Vm* = *m*. This way, it is possible to assess how much the estimated value of ***P*** deviates from the expected (details in Methods). No. ind. = Number of individuals.

### Temporal distribution of *O*. *suberosus* on La Escobilla

**Fig 4:**
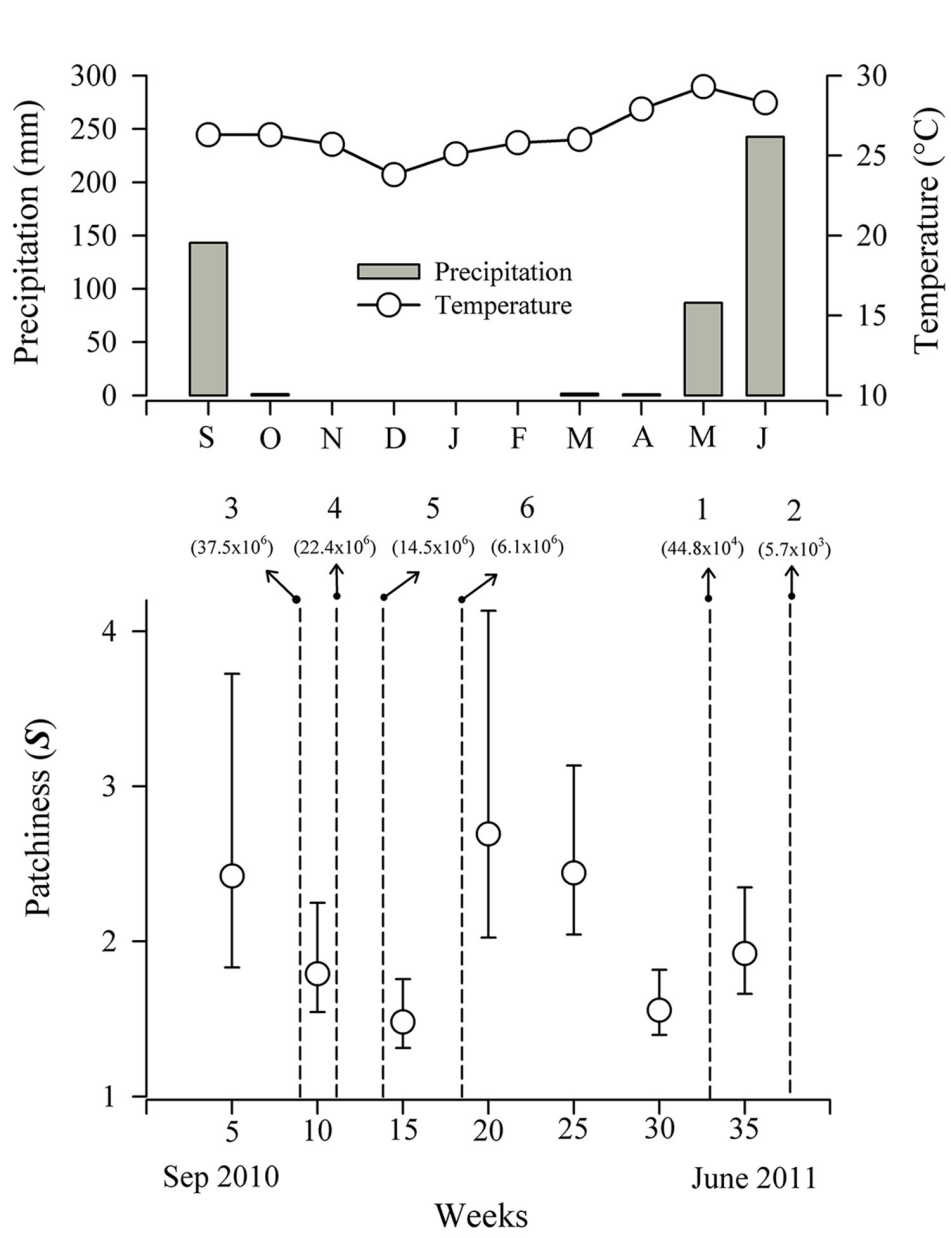
Patchiness Values (*S*) for adult *O*. *suberosus* over time (35 weeks). Also shown are the precipitation (mm) and temperature (°C) profiles for September 2010 to June 2011, when sampling was conducted (data supplied by the Comisión Nacional del Agua, CONAGUA, Mexico), from the Barra de Cozoaltepec climatological station, which is the one nearest to La Escobilla Beach. The numbers above the dashed lines indicate the final four *L*. *olivacea* arrival events of the 2010–2011 season and the two first arrivals of the 2011–2012 season. The estimated number of turtle eggs deposited on the beach during each arrival event is given in parentheses (data provided by the Centro Mexicano de la Tortuga–CONANP, Mexico).

Regarding temporal distribution, we found: 1) fluctuations in the aggregated distribution (***S*** > 1) of the adult beetles following the arrival of the turtles on the beach, 2) these values decrease with the decreasing number of turtle eggs in each arribada and during the period when there were no turtle arrivals (between the end of one season and the start of the next), and 3) subsequently there was a trend of increasing *O*. *suberosus* presence in the first arrival of 2011–2012 (Fig 4).

### The effect of the condition and abundance of *L*. *olivacea* turtle eggs on damage by *O*. *suberosus*

#### Time to onset of damage to the clutch

**Fig 5:**
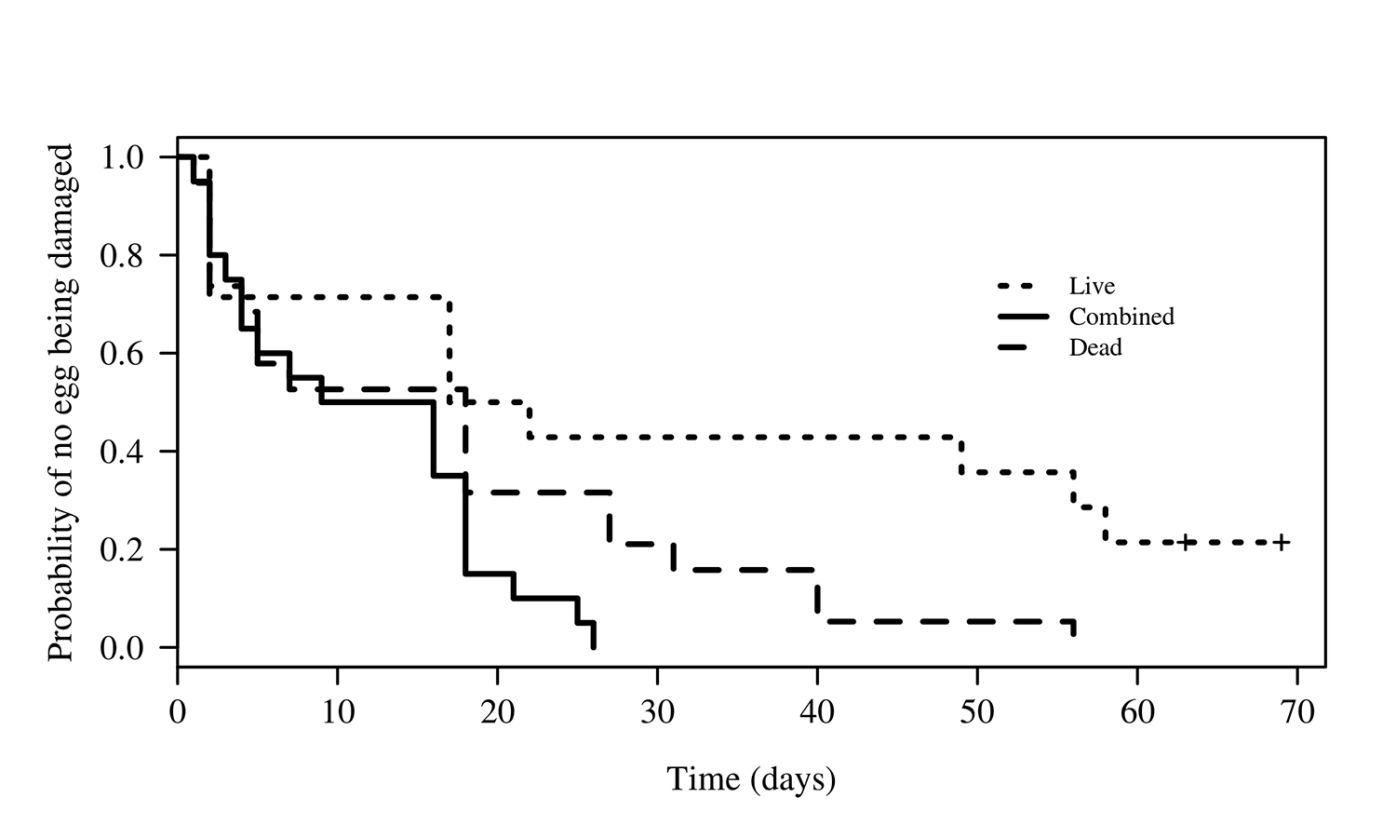
Time to first attack by *O*. *suberosus* on *L*. *olivacea* eggs, according to egg condition under laboratory conditions. + denotes eggs that were never attacked during the experiment. L = live eggs, C = combination of live and dead eggs, D = dead eggs.

Eggs had been incubating for one week at the beginning of the experiment with damage to the first egg occurring 2–17 days later. The minimum adequate model only included the condition of the egg as the sole factor associated with the probability that the beetle would begin to consume the turtle eggs. Egg abundance and the interaction between egg abundance and condition had no significant effect. Contrast analysis showed that the treatments with dead and live eggs (combined) were no different from those with only dead eggs (Z = 1.23, p = 0.22), but that the time to the onset of damage to the eggs in the treatments with live eggs was significantly longer than in the combined treatment (Z = 3.342, p = 0.0008, Fig 5), i.e., the treatments with both live and dead eggs were consumed more quickly by the beetles (mean ± SE = 11.5 ± 1.88 days, min. = 1, max. = 26 days). Live eggs were more likely to remain intact over time and more time elapsed before the beetles began to consume them (31.4 ± 7.11 days, min. = 2, max. = 69 days). The results for the treatments with dead eggs fell between those for live eggs and the combination of live and dead eggs (17 ± 3.70 days, Min. = 1, Max. = 56 days).

#### Time to damage of the entire clutch

**Fig 6:**
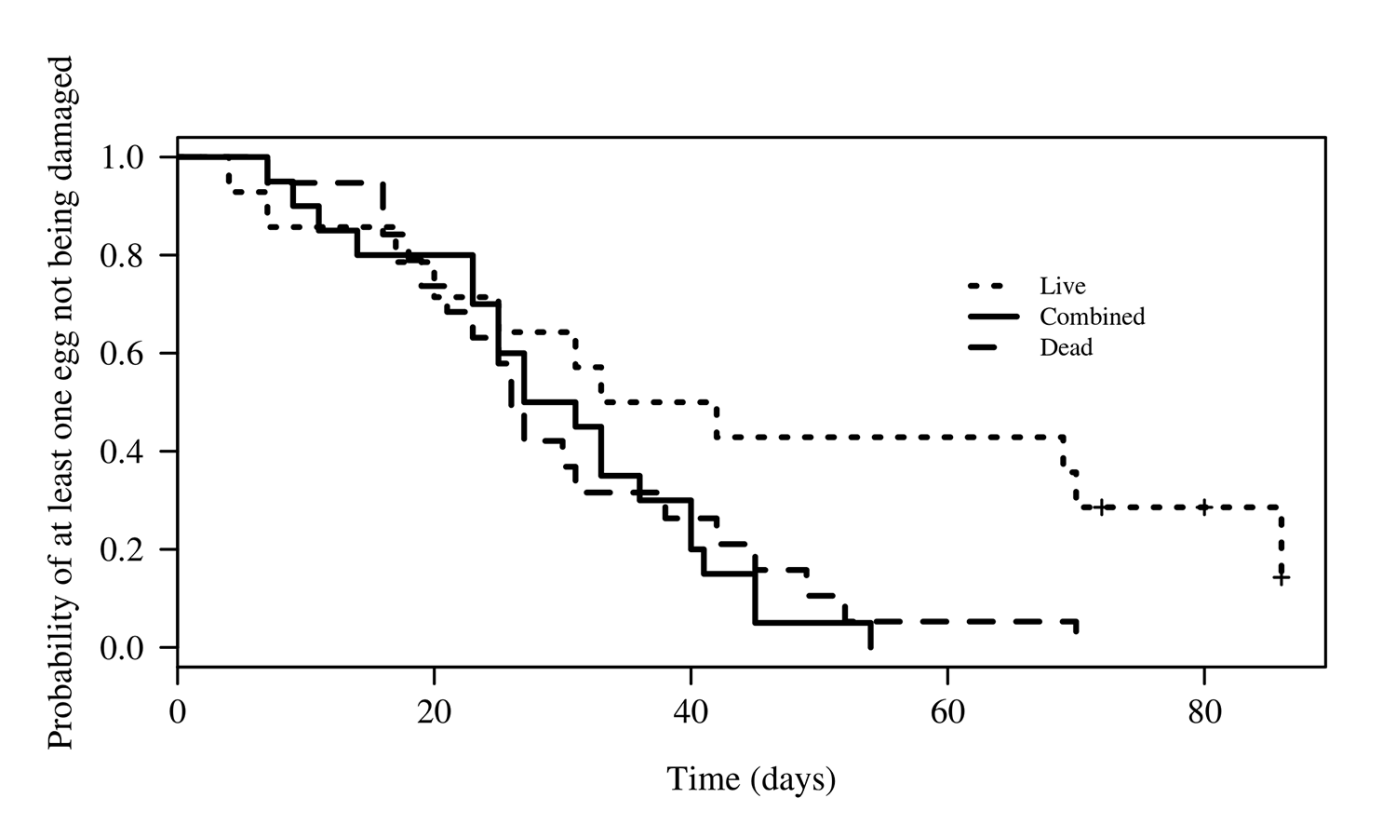
Time to total damage of the entire clutch of turtle eggs, as a function of egg type under laboratory conditions. + indicates eggs that were never attacked during the experiment. L = live eggs, C = combination of live and dead eggs, D = dead eggs.

The minimum adequate model for this variable included both egg abundance and condition, but the interaction between them was not found to be significant. The entire clutch of eggs was consumed more quickly when nest abundance low, although the difference was only marginally significant (Z = 2.02, p = 0.043). The mean time in days (±SE) for damage to the entire clutch for high nest abundance was 41.07 ± 19.7 (n = 26, min. = 18, max. = 86) and 27.55 ± 19.22 for low nest abundance (n = 27, min. = 4, max. = 72). The contrast analysis detected no difference in the time to total damage of the clutch between the treatments with both live and dead eggs (combined) and those with dead eggs only (Z = 0.368, p = 0.713); however, there were differences between the treatments with combined eggs and those with only live eggs (Z = 3.385, p < 0.001; Fig 6). Mean time in days (± SE) for total damage to clutches with dead eggs was 30.58 ± 3.52 (n = 19, min. = 7, max. = 70) compared to 45.86 ± 8.05 (n = 14, Min. = 4, Max. = 86) for nests with live eggs. Nests with both live and dead eggs took 29.45 days ± 2.88 (n = 20, min. = 7, max. = 54) to be completely consumed.

### Life cycle

We found *O*. *suberosus* eggs and all stages of its immature phase buried in the sand and close to the turtle eggs. The *O*. *suberosus* eggs and larvae were easily found due to their coloring: white and cream, respectively. The complete life cycle of *O*. *suberosus* lasts a mean of 51.7 days (min. = 35, max. = 71). Mean duration (± SE) of each development stage was: egg to 1^st^ instar larva: 5.6 ± 3.7, 1^st^ to 2^nd^ instar larva: 7.3 ± 4.5, 2^nd^ to 3^rd^ instar larva: 10.0 ± 4.2, 3^rd^ instar larva to pupa: 15.0 ± 4.0 and from pupa to first adult: 13.8 ± 2.2 days. We also found that each female laid a maximum of four eggs.

## Discussion

This exploration of the relationship between the frequency of *O*. *suberosus* and the proportion of damaged eggs in the nests of *L*. *olivacea*, the spatial and temporal distribution of the beetles and the probability of damage to the turtle eggs under experimental conditions produced some interesting findings. We noted that the population dynamics of the beetles seems to vary with the arrival pattern of the turtle; in both seasons, the frequency of adult beetles influenced the frequency of larvae (Fig 3), but this also seemed to be independent of the density of turtle nests. However, at the beginning of the 2011–2012 season, larvae were very scarce (none at low nest density and only seven at the high density) compared to adult beetles. Our findings support the observation of M. Sánchez–Carrillo (pers. comm.) given that differences between the immature and adult stages found in the two arrival seasons seem to suggest that the reproductive cycle of *O*. *suberosus* females is synchronized with the arrival of the turtles.

Our results show that the presence of the beetle in the nests of the turtle and their consumption of eggs on the beach is a complex phenomenon related to the dynamics of resource availability (i.e., the abundance and quality of eggs) during arrivals and to the life history traits, reproductive biology and population demographics of *O*. *suberosus*. This makes it difficult to interpret of some of the results. Thus, we believe it is important to keep in mind that the aim of this study was only to compare the potential impact of the beetle on turtle eggs in contrasting densities (high and low) for two different arrivals, which turned out to be quite different in the amount of eggs available on the beach during each one (Table 1). This reflects the tremendous spatial and temporal variation in the density of nests along the beach and the limitations of sampling at detecting this variation. For example, similar clutch size in high nest density 2008–2009 and low nest density in 2011–2012 could be explained by the greater concentration of nests on smaller parts of the beach during the latter.

In the 1^st^ arrival of 2011–2012, which was one of the shortest of the season (2 days), for both nest densities we found that all of the eggs appeared to have been damaged by adult beetles that complete their life cycle in these nests, since no immature stages were found, and that the vast majority of eggs had perforations typical of those produced by *O*. *suberosus*. The complete damage suggests that the beetles consumed all of the eggs when this food resource was less abundant, since during the season 2008–2009 the higher proportion of damaged eggs was also found at low nest density (Fig 2). These results did not support our prediction of greater egg damage at the higher nest density.

One of the classic hypotheses used to explain why olive ridley turtles nest in mass events is that with this strategy some eggs can escape predation [14]. Given that the 1^st^ arribada of 2011–2012 was one of the smaller ones of the season (fewer than 60,000 eggs; see Fig 4), the intense nest predation by the beetles would be consistent with that hypothesis. That is, with fewer eggs available (and probably a greater concentration of eggs) the beetles might have had a greater opportunity to consume the eggs and complete their life cycle more quickly. Equally, the scarcity of larvae in the completely damaged nests during this season suggests that *O*. *suberosus* females do not necessarily depend on live turtle eggs to oviposit, thus supporting the idea that this beetle species, rather than a predator, could be recycling organic material on La Escobilla.

The hatching success of the olive ridley turtle depends on a complex interaction between biotic (i.e., predation, microbial attack) and abiotic (i.e., temperature and humidity) factors around the nest, which affect embryonic development [1]. High nest densities and high rates of nest destruction are associated with mass nesting behavior, which increases the supply of organic material [14]. At La Escobilla, like other beaches, there are temporal and spatial fluctuations in food supply (more than 6 million eggs per season, representing organic material calculated at 2 tons per year on this beach; Sanchez–Carrillo, pers. comm.); fluctuations that not only dictate the aggregation patterns of beetles on the beach, but also their reproductive cycle. However, it is important to note that our sampling design does not reflect the spatio–temporal dynamics of the arribadas on the beach and that may introduce a potential source of bias, such as variation in the number of nests within each of the areas selected as being of high and low density [7].

We would like to highlight several aspects of the spatial and temporal distribution of *O*. *suberosus* on La Escobilla. First, our results do not support the prediction that the beetles would only be aggregated at a low density of turtle nests due to the lower availability of food. Second, considering that the index of spatial patchiness (***P***) is one that best expresses biological behavior [26, 37, 43], our results suggest that the beetles appear to have been competing more intensely in sites with low nest density than in high density sites at the beginning of the 2011–2012 turtle arrival season, when the resource was scarce. This is supported by the higher spatial patchiness (***P***) values of the adult beetles (Table 2), which fits the pattern observed on the beach, since most individuals of *O*. *suberosus* were grouped into a few nests (or patches). Hence, the beetles at low nest density are likely experiencing a higher level of crowding, which was a bit more than double (2.6) that found at the high nest density during the same season. Conversely, values of spatial patchiness were lower at high nest density for both arrival seasons (Table 2A and 2B). This seems to suggest a reduction in competition for this resource as a result of an increased availability of food, as we found a greater number of nests with fewer beetles, which may explain why the proportion of damaged eggs was smaller (Fig 2). Since we do not have a full understanding of the way in which changes in nest density on the beach affect the spatial and temporal distribution of the beetle, our results offer a baseline from which to design future studies.

The most interesting result was that of the fluctuating behavior of the temporal aggregation values (***S*** > 1, in all cases) for this beetle in relation to the arrivals of female olive ridley turtles on this beach (Fig 4). The observed fluctuation could be related to the synchronization of the reproductive cycle of the beetle with the pattern of turtle arrivals, a dynamic that seems to respond to environmental conditions, since the larger arribadas do not occur until the rains diminish (Fig 4). Our results demonstrate that the population of *O*. *suberosus* on La Escobilla beach is not occasional, but rather is permanent and appears to behave as a multivoltine species (M. Sánchez–Carrillo, unpubl. data), with an aggregated distribution that fluctuates between the turtle arrivals (Fig 4). These results point the way for future reproductive behavior studies, comparing the reproductive patterns of the beetle with *L*. *olivacea* arrival events, aspects that are of great interest given that the mating and reproductive strategies of these insects are still unknown and may be influenced by resource availability.

The results obtained under laboratory conditions demonstrate that, while *O*. *suberosus* did consume dead eggs, it was also capable of eating live eggs, confirming our observations on the beach. We wish to highlight the differences in the time taken to consume the eggs by the beetles, which leads us to propose two possible explanations. The first is based on the fulfilled prediction that the beetles would more rapidly consume dead (17 days) and combined dead and live (11 days) eggs than they would consume live (31 days) eggs (Figs 5 and 6). Since this species is efficient at removing organic carbon [4], we propose that, at least for La Escobilla, this population of beetles could be recycling the large quantity of decomposing organic material (i.e., dead turtle eggs, embryos, juveniles and adults) that accumulates within the nests and on the surface of the beach during each arrival [44]. Extrapolation of these results to beach conditions could explain the greater attack on the eggs in the area of low density in the 2008–2009 season.

Under natural conditions, many nests have a combination of live eggs with evident development (no perforations) and dead eggs (with perforations) in an advanced state of decomposition, as well as a great quantity of egg shells, apparently the result of intra–specific nest destruction [8]. This modifies the micro–environmental conditions (i.e., higher CO_2_ concentration and increased temperature) within the nests as a consequence of microbial activity [45], which can facilitate the rapid consumption of the eggs by the beetles. The increased temperature within the nests may explain the greater feeding and reproductive behavior of this beetle, as reported for two species of *Omorgus (O*. *asperulatus* Harold and *O*. *freyi* Haaf), species that reach their reproductive optimum at temperatures close to 34°C [46]. This represents a favorable situation for this necro–saprophagous beetle and also for the flies and other beetle species that were observed in abundance in the turtle nests, and which may be another factor affecting the survival of this turtle species.

Given the evidence presented in this study that *O*. *suberosus* also eats live eggs, another hypothesis is that *O*. *suberosus* potentially behaves as a prejudicial species in *L*. *olivacea* nests. Our observations support this since the 46 days this beetle took to consume all the live eggs under laboratory conditions would be sufficient time to interrupt the natural incubation period of the turtle, which lasts around 49 days on La Escobilla [47] and up to 60 days on Nancite Beach [44]. This could have a direct effect on eclosion success, which has decreased in recent years from the typical level of 30% for the La Escobilla turtles to 5% and 10% [3, 7, 44, 47]. However, the magnitude of the impact of *O*. *suberosus* on the survival of *L*. *olivacea* and other species of sea turtle is still not clear. Further research is necessary to test these two hypotheses (beetles as organic matter recyclers, or as egg pests).

Another factor to consider is that the complete life cycle of *O*. *suberosus* under laboratory conditions lasts an average of 52 days. Given that both the larvae and adult beetles consume eggs, any stage could interrupt the incubation period of the turtle eggs on La Escobilla. The simultaneous presence of all developmental stages observed in both the field and the laboratory confirms that *O*. *suberosus* quickly develops to maturity and this allows us to infer that several generations overlap throughout the year on La Escobilla. The rapid development of these beetles raises the possibility of a rapid response in local beetle populations to the temporal dynamics of the arrival *en masse* of the sea turtles, as confirmed by the variation in the beetle aggregation values and patchiness on the beach. If this is indeed the case, then there should be more *O*. *suberosus*, especially larvae, two or three weeks after each wave of laying turtles arrives. According to our results, the life cycle of *O*. *suberosus* is relatively short and is similar to that reported by Scholtz [48], which contradicts the suggestion of Lago *et al*. [49] that the life cycle of *O*. *suberosus* lasts one year.

Another laboratory result derived from the effect of turtle egg abundance on the probability of damage by *O*. *suberosus* revealed that both the initial stage of damage and total consumption of eggs occurred more rapidly (27 days and 14 days before the end of the experiment, respectively) when egg abundance was low than when it was high. This result, which is an indirect measurement of nest density, along with the fact that the beetles were more aggregated in areas of low nest density on La Escobilla, suggests that at this density the beetles can damage the turtle eggs in a shorter period of time. The higher proportion of damaged eggs found at low nest densities does not seem to concur with the explanations provided to date, namely that turtle survival can be affected by this beetle to a greater extent at high nest densities [2, 5–6, 34]. Our field and laboratory results regarding the time of consumption of eggs by *O*. *suberosus* show that the condition and abundance of these eggs are factors that must be taken into account in future studies.

The limitations to evaluating the effect of turtle nest density on the impact of egg damage by *O*. *suberosus* under laboratory conditions (i.e., the need to periodically acquire permits for extracting eggs from the beach, which is a protected area for turtles, as well as the logistical difficulty of replicating and simulating conditions similar to those of the beach, among others) may lead other researchers to focus their questions on different aspects of this phenomenon. For example, they may wish to explore whether there are variations in the reproductive behavior of the beetles that follow the arrival events of *L*. *olivacea*, or evaluate the survival of *L*. *olivacea* as a function of the levels of aggregation of *O*. *suberosus* on La Escobilla. An assessment of whether the levels of damage caused by *O*. *suberosus* are sufficiently high for the beetle to be considered a pest of the *L*. *olivacea* eggs would be helpful. Experimental manipulation is required to directly test the impact of these beetles on the survival of *L*. *olivacea*’s offspring. However, indirect tests such as those employed in this study continue to be both valuable and necessary for the planning of future studies on this topic and for the conservation of this marine turtle.

### Management and conservation implications

The results of this study confirm that in one of the most important massive nesting areas of the sea turtle *L*. *olivacea*, the beetle *O*. *suberosus* could be another mortality factor for the populations that nest along the eastern Pacific coast. While this beetle is considered a facultative necro–saprophage, it can also feed on live eggs. The observed synchrony between the reproductive cycle of the beetle and the dynamic of the turtle arrival events over the course of each nesting season suggests that this beetle could be a risk factor for the eclosion of the hatchlings, affecting recruitment into the population. We therefore think it is necessary to: 1) determine whether *O*. *suberosus* is present on other massive nesting beaches along the coast, in order to gain a regional overview of this marine turtle’s geographic distribution, 2) document the local fluctuations in beetle abundance during each nesting season, and 3) evaluate the magnitude of the damage caused by the beetle to the turtle eggs in areas with different nesting densities. These three proposals require the concerted efforts of the authorities and research institutions, including NGOs interested in the conservation and management of *L*. *olivacea*. The information obtained from several beaches would allow for the timely implementation of suitable methods for controlling populations of *O*. *suberosus* if necessary.

## Supporting Information

S1 Table Captures of *O*. *suberosus* (adults and larvae) in quadrats and nests on La Escobilla Beach for two arrival seasons (2008–2009 and 2011–2012). (XLS)

S2 Table Captures of *O*. *suberosus* in traps with chicken feathers over 35 weeks on La Escobilla Beach. (XLS)

S3 Table Data for calculating the probability of no egg being damaged. (XLS)

S4 Table Data for calculating the probability of at least one egg not being damaged. (XLS)
